# Association of HLA-DRB1 polymorphism with Alzheimer's disease: a replication and meta-analysis

**DOI:** 10.18632/oncotarget.21479

**Published:** 2017-10-04

**Authors:** Rui-Chun Lu, Wu Yang, Lin Tan, Fu-Rong Sun, Meng-Shan Tan, Wei Zhang, Hui-Fu Wang, Lan Tan

**Affiliations:** ^1^ Department of Neurology, Qingdao Municipal Hospital, Qingdao University, Qingdao, China; ^2^ International Department, The Affiliated Hospital of Qingdao University, Qingdao, China

**Keywords:** Alzheimer’s disease, polymorphism, association study, HLA-DRB1, meta-analyses

## Abstract

Genome-wide association studies (GWAS) have identified one single-nucleotide polymorphism (SNP) rs9271192 within *HLA-DRB1* as a risk factor for Alzheimer's disease (AD) in Caucasians. The effect of rs9271192 on AD needed to be verified in other ethnic cohorts. In order to evaluate the association between *HLA-DRB1* rs9271192 polymorphism and late-onset AD (LOAD) in the Northern Han Chinese population, we recruited 982 LOAD patients and 1344 sex- and age-matched healthy controls. The results showed that *HLA-DRB1* rs9271192 was associated with LOAD (genotype *P* = 0.015, allele *P* = 0.04). The results of logistic regression revealed the C allele homozygosity strongly increased the risk of LOAD under a recessive model in the total sample (*P* = 0.004, OR =2.069, 95% CI = 1.262–3.434). When these data were stratified by apolipoprotein E (*APOE*) ε4 status, the observed association was confined to APOE ε4 non-carriers (additive model: P=0.048, OR =1.191, 95% CI =1.001–1.417; recessive model: *P* < 0.001, OR = 2.601, 95% CI =1.519–4.566). Furthermore, meta-analysis after sensitive analysis confirmed that rs9271192 within *HLA-DRB1* increased the risk of LOAD (OR = 1.12, 95% CI = 1.08–1.15). To summarize, the C allele in *HLA-DRB1* rs9271192 may be an independent risk factor for LOAD.

## INTRODUCTION

Alzheimer's disease (AD) is a complex and multifactorial neurodegenerative disease [1, 2]. Recently, increasing researches show that heritability for AD is high [3, 4]. In a large twin study suggested that heritability for AD was estimated to be 79% in the best-fitting model [4]. So far, apolipoprotein E (*APOE*) was unequivocally demonstrated as established susceptibility gene for late-onset AD (LOAD) [5, 6], but the variation of *APOE* was not sufficient for the development of the disease. This suggest that there are additional risk loci influence the susceptibility of AD that remain to be discovered [7]. In recent years, Genome-wide association studies (GWAS) as genetic association studies have shed light on the genetic basis of LOAD, which can discover more several genes and/or loci involved in the susceptibility to suffer this disease [8, 9]. The meta-analysis of GWAS had been performed in LOAD and tagged *HLA-DRB1* rs9271192 as new susceptibility loci in Caucasians [10]. Since variants and their frequencies of *HLA-DRB1* gene in various ethnic groups might be different, further replication became the urgent task to be performed in other ethnic cohorts [7, 11–15]. Hence, we conducted an association analysis between rs9271192 SNP within *HLA-DRB1* and LOAD to verify the above conclusions in the Northern Han Chinese population. Furthermore, there were other two large-scale studies investigated an association analysis between rs9271192 SNP within *HLA-DRB1* and LOAD in Chinese, did not obtain a consistent conclusion in Chinese. So, we collected our data along with previously studies for meta-analysis to reach a more credible conclusion.

## RESULTS

### Replication study

There were no significant differences in gender and age (*P* = 0.067, *P* = 0.189, respectively) between AD and controls. Significantly lower Mini Mental State Examination (MMSE) score was found in LOAD patients compared to the controls (*P* < 0.001). As expected, the presence of the *APOE* ε4 allele was associated with of LOAD (*P* < 0.001) (Table 1).

**Table 1:** The characteristics of the study population

	AD (*n* = 982)	Control (*n* = 1344)	*P* value	OR (95% CI)
Age at examination, years; mean ± SD	79.83 ± 6.69	75.49 ± 6.48	0.189^*^	
Age at onset, years; mean ± SD	75.17 ± 6.08			
Gender, *n* (%)			0.067	
Male	408 (41.50)	596 (44.30)		
Female	574 (58.50)	748 (55.70)		
MMSE score, mean ± SD	11.94 ± 6.21	28.49 ± 1.09	< 0.001	
APOE ε4 status, *n* (%)			< 0.001	
APOE ε4 (+)	280 (28.60)	189 (14.10)		2.45 (2.00–3.01)
APOE ε4 (-)	702 (71.40)	1155 (85.90)		

Abbreviation: AD, Alzheimer's disease; Control, healthy controls; OR, odds ratio; CI, confidence interval; MMSE, Mini-Mental State Examination; APOE, apolipoprotein E; SD, standard deviation.

^*^P value was calculated with the age of onset for late-onset AD and age at examination for Control. Differences in the characteristics of age and MMSE score between the two groups were examined using Student's *t* test. Differences in gender and APOE ε4 frequency between AD patients and Control were assessed using the Pearson χ^2^ test.

Deviations from distributions of the polymorphisms were excluded by the HWE version 1.20 in both LOAD patients and controls. The allele and genotype frequencies of LOAD patients and controls in the total sample and after stratification for *APOE* ε4 allele were shown in Table 2. The rs9271192 polymorphisms in *HLA-DRB1* had significant differences in the genotype frequencies in the total sample (*P* = 0.015). And rs9271192 polymorphisms in *HLA-DRB1* reached significant differences in the allele frequencies in the total sample (*P* = 0.04). Higher frequencies of the minor allele (C) were observed in patients with LOAD compared with control subjects (19.04% vs. 16.74%). When the genotype and allele distribution were stratified by the *APOE* ε4 allele status, we observed significant differences in the genotype frequencies in the *APOE* ε4 non-carriers (*P* = 0.002). In *APOE* ε4 allele carriers, the genotype and allele distribution of rs9271192 between LOAD patients and controls was no significantly different (*P* = 0.704, *P* = 0.958, respectively). Based on the observed prevalence of the minor alleles in controls, our sample size had greater than 90% power to detect a relative risk of 1.2 for rs9271192.

**Table 2:** Distribution of the rs7294919 genotypes and alleles in AD cases and controls stratified by *APOEε4* presence

Total	*N*	Genotype			*P*	Allele		*P*
		CC (%)	CA (%)	AA (%)		C (%)	A (%)	
AD	982	40 (4.07)	294 (29.93)	648 (66)	0.015^*^	374 (19.04)	1590 (80.96)	0.04^*^
Controls	1344	28 (2.08)	394 (29.32)	922 (68.60)		450 (16.74)	2238 (83.26)	
APOEε4 (+)								
AD	280	6 (2.14)	96 (34.29)	178 (63.57)	0.70	108 (19.29)	452 (80.71)	0.96
Controls	188	6 (3.19)	60 (31.92)	122 (64.89)		72 (19.15)	304 (80.85)	
APOEε4 (−)								
AD	702	34 (4.84)	198 (28.21)	470 (66.95)	0.002^*^	266 (18.95)	1138 (81.05)	0.86
Controls	1156	22 (1.90)	334 (28.89)	800 (69.21)		378 (16.35)	1934 (83.65)	

Notes: AD, Alzheimer's disease; APOE ε4 (+) subjects who had 1 or 2 ε4 alleles; APOE ε4 (−) subjects who did not have the ε4 allele; CI, confidence interval; OR, odds ratio; P, *P* value.

^*^ Mean *P* ≤ 0.05.

We further investigated the distributions of the rs9271192 polymorphism in multivariate logistic regression analysis (adjusted for age, gender, and the *APOE* ε4 allele status for the total sample; adjusted for age and gender for the subsets) (Table 3). The results revealed C allele homozygosity strongly increased the risk of LOAD under a recessive model in the total sample (*P* = 0.004, OR = 2.069, 95% CI = 1.262–3.434). We further evaluated the association of rs9271192 with LOAD risk in patients with or without *APOE* ε4 allele using logistic regression in order to rule out confounding factors in the subsets. As indicated by Table 3, in non-*APOE* ε4 carriers, the C allele at rs9271192 increased LOAD risk in additive model and recessive model (additive model: *P* = 0.048, OR = 1.191, 95% CI = 1.001–1.417; recessive model: *P* < 0.001, OR = 2.601, 95% CI = 1.519–4.566).

**Table 3:** Logistic regression analysis of rs9271192 SNP in *HLA- DRB1* gene

SNP	Group	Model	OR (95% CI)	*P*
rs9271192	Total^a^	Add	1.147 (0.981–1.340)	0.085
		Dom	1.088 (0.910–1.301)	0.355
		Rec	2.069 (1.262–3.434)	0.004^*^
	APOEε4 (+)^b^	Add	0.992 (0.702–1.407)	0.962
		Dom	1.042 (0.707–1.542)	0.834
		Rec	0.629 (0.193–2.048)	0.430
	APOEε4 (−)^b^	Add	1.191 (1.001–1.417)	0.048^*^
		Dom	1.105 (0.903–1.350)	0.332
		Rec	2.601 (1.519–4.566)	< 0.001^*^

Notes: CI, confidence interval; OR, odds ratio; P, *P* value; Add, additive model; Dom, dominant model; Rec, recessive model;

a Adjusted for age, gender, and the carriage of at least one *APOEε4* allele.

b *APOEε4* (+)/*APOEε4* (−) subset: adjusted for age and gender.

^*^ Mean *P* ≤ 0.05.

### Meta-analysis

**Figure 1:**
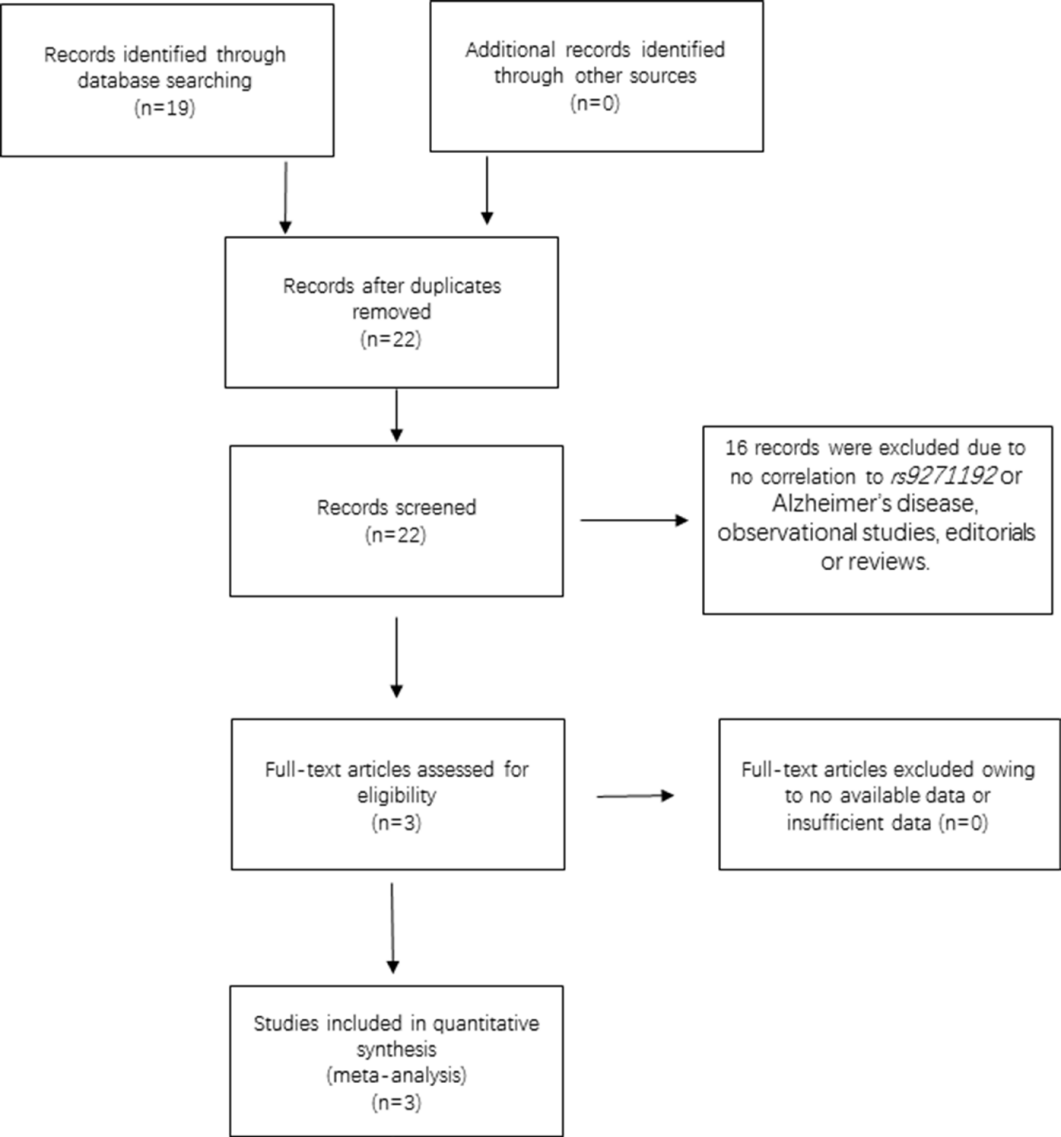
Flow chart of the search strategy and study selection

**Figure 2:**
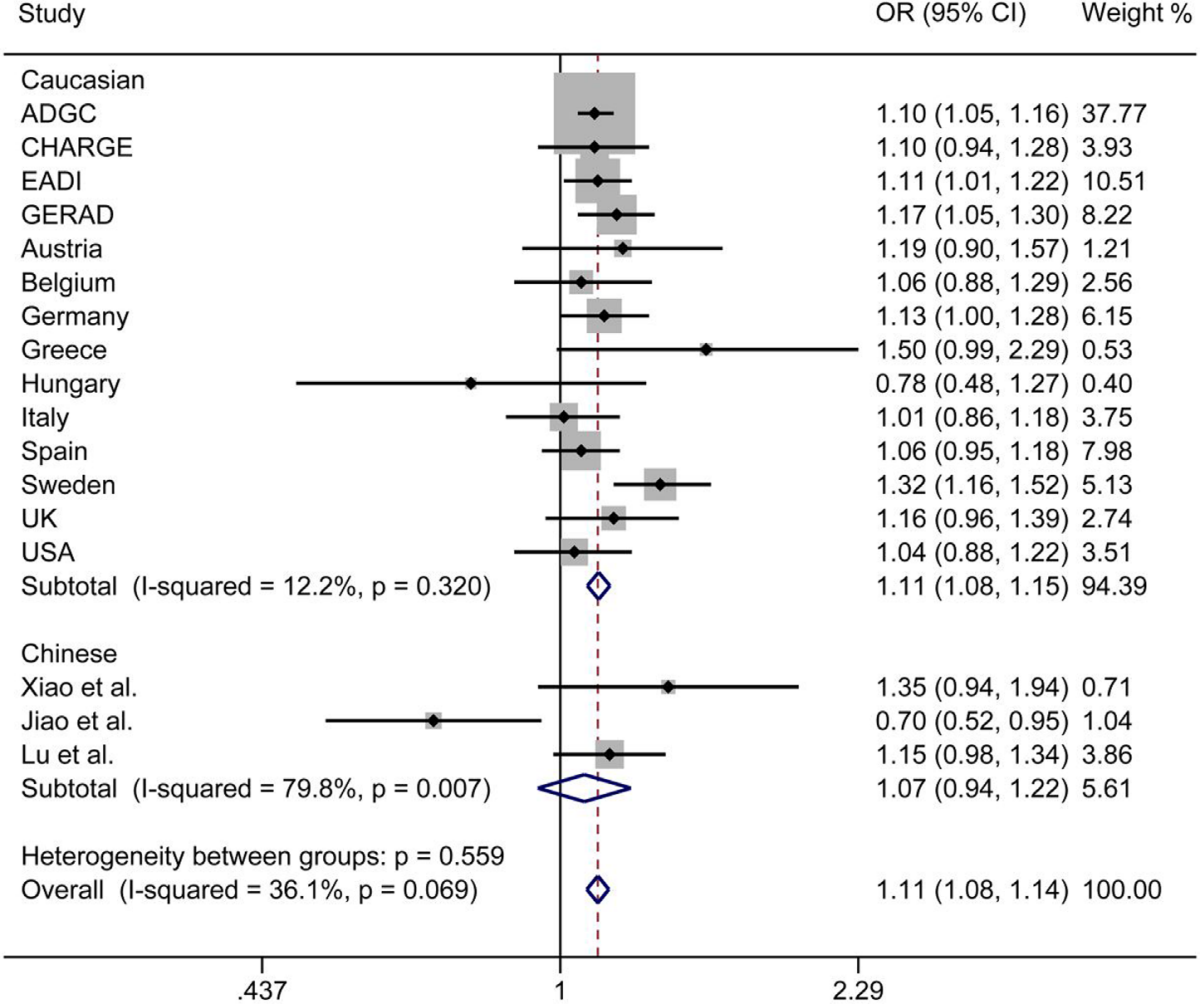
Forest plots for rs9271192 in LOAD and healthy controls in 82501 individuals, which show the association by ethnicity There was a high heterogeneity in the Chinese subgroup (I^2^ = 79.8%). OR: odds risk, CI: confidence interval.

**Figure 3:**
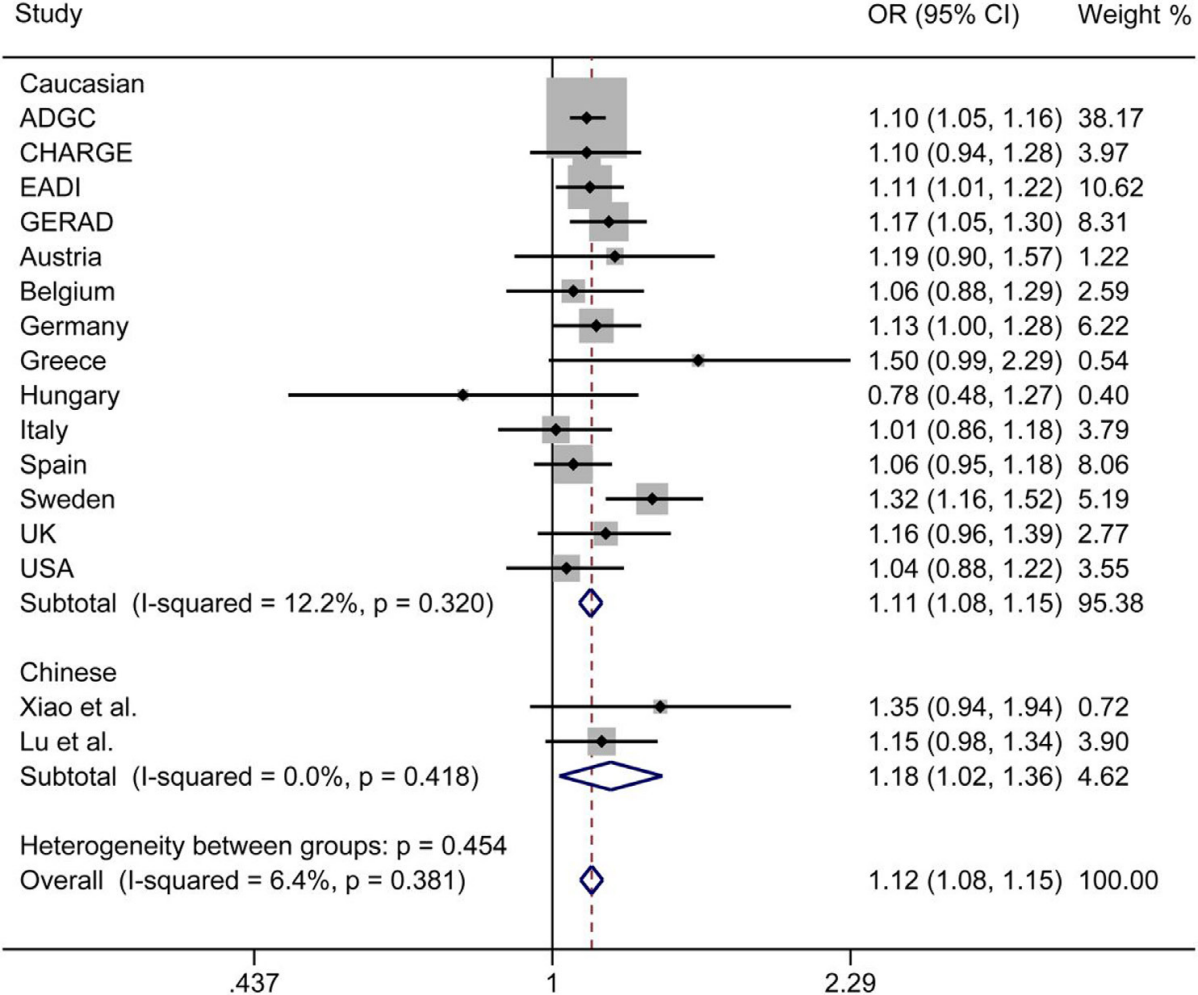
Forest plots for rs9271192 in LOAD and healthy controls in 81954 individuals, which show the association by ethnicity The heterogeneity significantly declined when excluding the Jiao et al. study. OR: odds risk, CI: confidence interval.

The initial search on computerized databases screened 19 articles. After 16 articles were excluded due to no correlation to rs9271192 or Alzheimer's disease, observational studies, editorials or reviews, 3 eligible articles were found (Figure 1). We extracted the data of different populations from the three large studies [9, 16, 17] and ours, then regrouped them according to Caucasians and Chinese as subgroup for meta-analysis. We found there was a high heterogeneity (I^2^ = 79.8%) in the Chinese subgroup (Figure 2). Thus, we carried out the sensitivity analysis and found that the heterogeneity significantly declined when excluding the study of Jiao et al. [17]. There is no obvious heterogeneity (Caucasians subgroup: I^2^ = 12.2%, Chinese subgroup: I^2^ = 0.0%) in the two subgroups (Figure 3). Finally, we found the less frequent allele (C allele) at rs9271192 was risk factor for LOAD in Caucasians (OR = 1.11, 95%CI = 1.08–1.15) and Chinese (OR = 1.18, 95% CI = 1.02–1.36) (Figure 3). Notably, our study found rs9271192 showed significant association with LOAD in pooled populations (OR = 1.12, 95% CI = 1.08–1.15) (Figure 3) without evident analysis heterogeneity (I^2^ = 6.4%).

## DISCUSSION

In our current study, there was statistically significant evidence for the *HLA-DRB1* genotype CC as risk factors to LOAD in the Northern Han Chinese population (recessive model: *P* = 0.004, OR =2.069, 95% CI = 1.262–3.434). Moreover, we also found that non-*APOE* ε4 carriers with rs9271192 genotype CC had a significantly higher risk of LOAD than those with the genotype CA+AA (recessive model: *P* < 0.001,OR = 2.601, 95% CI = 1.519–4.566). In contrast, *APOE* ε4 carriers with rs9271192 genotype CC did not have a higher risk than the other *APOE* ε4 carriers (recessive model: *P* = 0.430, OR = 0.629, 95% CI = 0.193- 2.048). The possible interpretation is that the genetic effect of *HLA-DRB1* is predisposing factor to LOAD in the absence of the *APOE* ε4 allele, while *APOE* ε4 allele is the most susceptible genetic factor in *APOE* ε4 carriers [18].

Furthermore, the effects of some genetic variants confirmed by GWAS in various ethnic groups might be different, due to population-specific and some unknown gene-gene or gene-environment interactions. To avoid these possibly complicated reasons and further investigate these associations, meta-analysis was performed in Caucasians and Chinese. We found there was a high heterogeneity (I^2^ = 79.8%) in the Chinese subgroup. There were two following researches made different conclusions in Chinese [16, 17]. Xiao et al. found no significant association between rs9271192 and AD (OR = 1.30, 95% CI = 0.91–1.85, P = 0.144). Jiao et al. drew diametrically opposite conclusion that C allele in *HLA-DRB1* rs9271192 on AD was found to be associated with decreasing LOAD risk (OR = 0.703, 95% CI = 0.521–0.949, *P* = 0.021). The different sample sizes, many unknown demographic, clinical variables and possibility of false positives might attribute to the different results. When we carried out the sensitivity analysis and excluded the study of Jiao et al. [17], the heterogeneity significantly declined (I^2^ = 0.0%). Finally, the results of meta-analysis showed that the C allele in *HLA-DRB1* rs9271192 may be an independent risk factor for LOAD, and the result coincided with the recent large-scale GWAS and our study.

The SNP rs9271192 locate at the 20917bp site upstream of the transcription start point of the *HLA-DRB1.* And the *HLA-DRB1* has a highly polymorphic region located on chromosome 6 associated with immunocompetence and histocompatibility, which is responsible for numerous immune responses [19]. Meanwhile, immune activation and possibly inflammation in the brain could play a remarkable role in the pathogenesis of AD were highlighted in numerous reports [20, 21]. Moreover, recent large-scale assessment of genetic risk factors associated with Parkinson's disease (PD) identified *HLA-DRB5* as novel risk loci [22]. PD and AD both are proteinopathy which characterized by neurodegeneration resulting from abnormal protein aggregation [23]. Given the association of this locus with PD, *HLA-DRB1* may also have a similar role in inflammatory responses that contribute to AD. All the above, we could hypothesize that inflammatory mechanisms could contribute to the pathophysiology of AD. However, Yu et al. had found that the methylation of *HLA-DRB5* was only nominal association with pathological AD diagnosis [24]. Hence, further biology evidence of *HLA-DRB1* from independent studies need to be warranted.

In summary, our current study has provided a convincing statistical support for an association between the *HLA-DRB1* polymorphism and LOAD, the carriage of C allele of the rs9271192 is associated with increased risk of LOAD in a Northern Han Chinese population. In the future, more studies in more large cohorts and in other ethnic groups are needed to validate the role of rs9271192 in LOAD. Furthermore, the additional independent replications and functional genetic analyses should elucidate the potential pathological mechanisms and the epidemiologic relevance of *HLA-DRB1* gene in AD.

## MATERIALS AND METHODS

### Subjects

Our study investigated 2326 subjects including 982 LOAD patients (mean age at onset: 75.17 ± 6.08 years; 584 women) and 1344 healthy control subjects (mean age at examination: 75.49 ± 6.48; 748 women) matched for gender and age. All the subjects in our study were unrelated Northern Han Chinese residents from Shandong province. The patients were assembled from the Department of Neurology of Qingdao Municipal Hospital and several other hospitals in Shandong. The subjects in case group were clinically diagnosed as “probable AD” by at least 2 neurologists, which according to the criteria of the National Institute of Neurological and Communicative Disorders and Stroke and/or Alzheimer's Disease and Related Disorders Association (NINCDS/ADRDA) [25]. The AD patients had no family history of neurodegenerative disorders or other dementias that were recruited in the case group. The control group was collected from the Healthy Examination Center of the Qingdao Municipal Hospital and at least two neurologists confirmed them healthy and neurologically normal by medical history, general examinations, laboratory examination, and MMSE score ≥ 28. The informed consent of this study was acquired from all subjects or their guardians, and our study was carried out with approval by the Institutional Ethics Committees. Our study was conducted with approval from the Ethical Committee of Qingdao Municipal Hospital.

### Genotyping analysis

Genomic DNA was extracted from the peripheral blood leukocytes by standard procedures using the Wizard genomic DNA purification kit (Cat. #A1125, Promega, USA). Genotyping for the *HLA-DRB1* (rs9271192) was carried out by a patent-pending technology of SNP scan which was developed on double ligation and multiplex fluorescence PCR from Genesky Biotechnologies Inc. Randomly selected DNA samples from each genotype were sequenced to validate the genotyping by ligation detection reaction method, and the case status of study subjects was blind to the laboratory staff. Results of the ligation detection reaction method corresponded with the results of sequencing.

### Statistical analysis

HWE version 1.20 (Columbia University, New York, NY, USA) was used to exclude deviations from Hardy–Weinberg equilibrium (HWE). Differences in the characteristics of the study subjects between the two groups were examined using the Student t test or the Chi-square test. Genotype and allele distributions were compared in the two groups by using the x^2^ test. A binary logistic regression model, adjusted for age (age at examination for control subjects), gender and *APOE* ε4 status, was used to estimate ORs and the 95 %confidence interval (CI) for testing possible associations between SNPs and AD. We defined various genetic models as AA vs. (CA+CC) for dominant, CC vs. (CA + AA) for recessive, and CC vs. AA for additive. The statistical power was calculated by STPLAN 4.5 software.

### Meta-analyses

### Search strategy

We carried out a systematic literature search of MEDLINE, EMBASE and the Cochrane library for studies published in the period from January 1995 to January 2016 to investigate the association between the *HLA-DRB1* rs9271192 polymorphism and AD. The key search terms including *HLA-DRB1, HLA-DRB5–DRB1*, rs9271192, Alzheimer's disease, and AD, combined with Boolean operators as appropriate. There were no language restrictions in our research. The reference lists of relevant primary articles were the source of additional studies.

### Study selection

The adopted articles were in accord to the following criteria: (1) case–control studies design; (2) Patients were included if they met the NINCDS–ADRDA criteria for AD diagnosis; (3) data of the ORs with corresponding 95% CIs were available in the report or could be calculated. Reviews, editorials, articles without essential data, and papers focused on familial AD were eliminated. Additionally, we only selected the data from the most comprehensive report for the meta-analysis if there was more than one publication from the same population.

### Data extraction and quality assessment

Two reviewers cooperated to accomplish data abstraction which was blinded to the authors and journal. Discrepancies in the collected data were discussed with other team members or contact with original investigators. If consensus was not reached, a third reviewer decided the final result. Once data of the ORs with corresponding 95 % CIs could not extract directly, we sought them from the authors. We tried to seek the missing information and essential clarification from the original authors. According to the data of each qualified study, we extracted the ORs with corresponding 95 % CIs from logistic regression. The study quality was assessed with the Newcastle-Ottawa Quality Scale (NOS). Our studies were considered high-quality, with a score of at least seven points.

### Statistical analysis

We pooled our data with the results from meta-analysis of 82501 individuals [9] and other reports about *HLA-DRB1* (rs9271192) and LOAD [16, 17] by fixed-effects inverse variance-weighted methods. We also generated I^2^ estimates with evaluate the possible effect of study heterogeneity on the results (I^2^ < 50 %, mean a lack of heterogeneity among populations). Our studies used Stata V.12.0 to perform all the meta-analyses.
